# Primus inter pares effect in high schools

**DOI:** 10.3389/fpsyg.2024.1382062

**Published:** 2024-10-11

**Authors:** Denis Dizdarević, Robert Leskovar, Goran Vukovič

**Affiliations:** Faculty of Organizational Sciences, University of Maribor, Maribor, Slovenia

**Keywords:** primus inter pares effect, better-than-average effect, illusory superiority, Dunning-Kruger effect, overestimation

## Abstract

*Primus inter pares* effect or better-than-average effect is cognitive bias known as illusory superiority in which individuals overestimate their positive abilities and traits in comparison to others. Overestimation and bias are often accompanied with various dangers on a personal, organizational or even societal level. We investigated the presence of overestimation among high school graduates in Slovenia in areas of teamwork, interpersonal skills, emotional intelligence, problem solving, and decision making. Although overestimation was present in all areas, results have also shown indications of indecisiveness. Overestimation was highest in the area of interpersonal skills, which is more of a social skill area in comparison with decision making or problem solving. Individuals probably receive more feedback over the course of high school in decision making, problem solving, and teamwork than in interpersonal skills, as those can directly impact grades while interpersonal skills less so.

## 1 Introduction

Nietzsche (1910) wrote that convictions are more dangerous to truth than lies. Darwin (1871) similarly that self-confidence arises more often from ignorance than knowledge and that those who know little, positively claim that science will never solve a problem. It is called *illusory superiority* or *better-than-average effect*, a state of cognitive bias in which individuals overestimate their abilities or traits compared to others (Alicke et al., 1995). Codol (1975) characterized illusory superiority as a *primus inter pares effect*. *Primus inter pares* in Latin means *first among equals* and comes from the Roman Empire. Gaius Octavius Augustus, the first Roman emperor, was titled *primus inter pares* (Ward et al., 2016). The phrase is somewhat contradictory, as it suggests that all are equal and yet that someone is first, and therefore suits for describing illusory superiority. *Illusory superiority* or *primus inter pares effect* as a form of cognitive bias is known as Dunning-Kurger effect, a cognitive bias where people with low abilities overestimate their abilities in a certain task and usually high performers underestimate their own skills (Kruger and Dunning, 1999; Dunning et al., 2003; Dunning, 2011). On the contrary, in an area where specific competencies are needed that are unknown to individuals, e.g., in the mechanics or construction of a spacecraft, most people underestimate their skills and a *below-average effect* occurs (Kruger, 1999). The *better-than-average effect* is due to an information deficit, the double curse of incompetence, uncertain lessons of feedback, poor definition of the nature of competencies, neglect of information, self-emphasis by neglecting others (egocentrism), and manageability of traits (Dunning et al., 2004). People are not aware of their shortcomings in social and intellectual abilities, and because individuals are unable to successfully complete a metacognition task, they consequently misjudge the success of the task performed. It is the aforementioned double curse phenomenon. In other words, they do not know that they do not know. Low performers firstly fail in tasks and make mistakes because they lack the competencies; secondly, they do not realize their errors due to their knowledge and skills gap (Kruger and Dunning, 1999; Dunning et al., 2003; Dunning, 2011). We can argue of course, that to some extent it is natural for one to overestimate himself in terms of motivation, plain survival, and everyday functioning. As also Pazicni and Bauer (2014) mention that excessive self-confidence is not necessarily always harmful, because self-efficacy is an important part of motivation, it can maintain motivation and consequently lead to success. However, we would add that one should be able to assess his own competencies as accurate as possible when making decisions. If people cannot accurately assess their own abilities, knowledge, traits, and cannot see their limitations, it can lead to bad and potentially dangerous decisions on personal level, organizational level, or even on the level of entire society. On a personal level, the consequences can be inadequate preparation for the exam; wrong choice of study; incorrect self-diagnosis of health; danger in traffic due to bad and wrong decisions, etc. On the level of entire society, consequences can be vast, as Machiavelli (2014) wrote, people often wish to change the government without any further plan or policies. They are simply unhappy with certain things, and based on that, they want a change at all costs, thinking that change will be for the better. In majority it turns out that is not the case and the situation actually worsens.

Both *primus inter pares effect* (or *better-than-average effect*; *illusory superiority*) and *Dunning-Kruger effect* are present across various domains, which we will cover in the next chapter. The aim of this paper is to discover the presence of *primus inter pares* effect and its degree among Slovenian high school graduates.

Alicke et al. (1995) state that the tendency to judge oneself more favorably than others can be seen as a heuristic that serves adaptive functions such as maintaining self-confidence (look at Study 7). People evaluate themselves more favorably when compared to others, but are less biased when compared to a specific person than generally compared to others, e.g., with the average student. This bias is further reduced if the individual has had contact with the person he is comparing himself to. This was also noted by Codol (1975) and Pedregon et al. (2012). Brown (2012) finds out in five studies that a better-than-average effect is greater for traits that participants think are more important.

In the domain of driving skills, a study that included participants from the USA and Sweden showed that most of the participants rated themselves as more skilled and safe drivers than others in the group (Svenson, 1981). Similarly drivers in the UK rated themselves as safer and more skilled than their peers and the average UK driver (Horswill et al., 2004). Applicants for a driving license in Finland and Netherlands assessed their driving skills, and candidates who failed the test largely overestimated their driving skills (Mynttinen et al., 2009). In pro-environment behavior domain, Bergquist (2020) conducted three studies with samples from Sweden, India, UK and, USA, in which the better-than-average effect was confirmed (study 1 does not show a dominant bias, but was also measured with one abstract question). The authors Mojzisch et al. (2021), researched the better-than-average effect of following the guidelines in the Covid-19 pandemic in UK, USA, Germany, and Sweden. Participants felt they adhered more strictly to the Covid-19 guidelines than others, and also that their friends adhered more strictly to the Covid-19 guidelines than the average citizen, but less than themselves. The results suggest a better-than-average effect, even in a pandemic, which is a relatively rare event in an individual’s life. Nowell and Alston (2007) find that higher self-confidence is shown in students with a lower average grade. Even in the domain of morality, the bias is present. Moral superiority has proven to be a very widespread and powerful form of positive illusion. Irrationality goes beyond what is observed in other areas of self-evaluation but is not related to self-esteem, as is typical elsewhere (Tappin and McKay, 2016).

### 1.1 Dunning-Kurger effect

Dunning-Kruger effect is a form of cognitive bias where people with low abilities overestimate their abilities in a certain task. The name originates from authors Dunning and Kruger, from their research in 1999. Participants from the first quartile overestimated their abilities the most. Their results put them in the 12th percentile, while they assessed their logical reasoning skills in the 68th percentile and their perceived test result in the 62nd percentile. Similarly, first-quartile participants overestimated their humor and grammar skills. Short training in the field of logical reasoning improved the identification of the limitations of their abilities (Kruger and Dunning, 1999). The most successful performers evaluate their work more accurately, while at the same time giving their peers overly optimistic performance assessments, which leads to modesty in their own self-assessment. They assume that others have a similar level of knowledge and skills as they do (Ehrlinger et al., 2008). Kruger and Dunning (1999) mention that it is a mathematical truth that people in the lowest quartile overestimate themselves, but it is important to what extent this deviates from their actual result. Key findings and implications of Dunning-Kruger effect are that poor performers overestimate their abilities because they do not know what they are doing poorly. They lack the skill or knowledge in certain topic or field and they lack metacognition.

A study from Netherlands found that low-skilled students rate their skills highly and high-skilled students more accurately (Feld et al., 2017). In a study from Russia, students who do not work rated their knowledge and skills as sufficient for a successful career start, while students who work were more critical (Akhmetshin et al., 2019). Pazicni and Bauer (2014) after multiple measurements, confirm the Dunning-Kruger effect among students in general chemistry programs. Also, students who overestimated themselves on the first exam did so later as well. Research among chemistry students conducted by Webb and Karatjas (2018) confirmed the bias, as did research by Bell and Volckmann (2011). The latter argue that for students who do not know much about the subject but are at the same time overconfident about their knowledge of the subject, the feedback they should gain from knowledge tests is largely a waste. The Dunning-Kruger effect was also confirmed in a study in the field of upper biology in the USA (Ziegler and Montplaisir, 2014).

In the study from Abu Dhabi, authors conducted a study, where success depends on the ability and will of the participants to get involved in analytical processing. The results showed that people who trust their intuition more also overestimate their abilities more (Coutinho et al., 2021). Bunay et al. (2017), in a study in Ecuador, measured skills of vocabulary, logical reasoning, and humor and confirmed the Dunning-Kruger effect. Saito et al. (2020) examined the Dunning-Kruger effect in the learning of a second language (English) by Japanese high school students. The study suggests a Dunning-Kruger effect. After a period of 3 months of study, during which participants learned the language, they calibrated their self-esteem more accurately. Participants who additionally practiced the language outside the syllabus calibrated their self-assessment more accurately over time.

Among aviation students from the USA, research showed the low performers were the ones that overestimated their results the most. The first part was in the field of grammar, and the second was in the field of examinations in the knowledge of the Federal Aviation Administration (FAA) (Pavel et al., 2012). Austin et al. (2008) explored the field of self-assessment ability of international pharmacy graduates, pharmacists from outside the USA and Canada seeking a license in Canada. Participants in the lowest quartile significantly overestimated their abilities. None from the bottom two quartiles met the minimum objective performance standards for pharmacists based on those tests. The limitation of the study is its relatively small sample. Dunning et al. (2004) stated that students tend to be overconfident in newly learned skills due to the educational practice of mass education, which encourages the rapid acquisition of skills and self-confidence but not the retention of skills. Coutinho et al. (2021) warned that the failure of individuals to recognize their lack of skills and knowledge in a particular field can lead to suboptimal study decisions, limits learning and performance, and makes it difficult to overcome ignorance and incompetence.

Martinussen et al. (2017) examined the accuracy of self-assessment of driving abilities of young drivers in Denmark. The results showed that participants’ self-assessment was inconsistent with their driving skills, especially in hazard perception. The authors point out that road accidents are probably one of the most negative feedbacks on driving skills, but because they are relatively rare, this leads to an overestimation of the competencies of both less and more capable drivers. As Weinstein (1987) wrote, they are most likely to provoke unrealistic optimism, that if a danger has not yet occurred, it is unlikely to happen in the future. This bias decreases with the frequency of personal experience and increases with the perceived preventability of a hazard. Dunning-Kruger effect was confirmed among the Canadian high school volleyball coaches (Sullivan et al., 2018). Tremayne et al. (2021) investigated whether the Dunning-Kruger effect can occur in the motor performance domain, where physical strength plays an important role. A study was conducted in Australia, all participants engaged in sports or physical activity at least three times a week. Cognitive bias was confirmed. A study in Slovenia by Plohl and Musil (2018) indicates that increasing the amount of information in the domain of nanotechnology but not the quality of that information increases false certainty in responses.

Errors in self-assessment can also cause challenges in the organizations in which the individual works. When a manager, superior, or expert has to mentor and advise others, success also depends on the knowledge and skills of the person receiving the advice. People resist negative feedback, looking for ways and reasons to reject it. Top performers believe that others also have the knowledge and skills they have. In a group or team, everyone quickly recognizes low performers and their mistakes, but on the contrary, at the highest level, the knowledge of the best individual exceeds the knowledge of the majority in the group, even of the other top performers. Other members of the group do not have the competencies to recognize the ability and knowledge of the best, so his decisions are recognized as potential mistakes (Dunning, 2015). Bias can also lead to bad and dangerous decisions at the level of entire societies. One such example is the anti-vaccination movement. Motta et al. (2018) conducted a study in the US and found that the people who showed the most excessive self-confidence were those with the least knowledge about the causes of autism and with misinformation about the link between vaccines and autism. These individuals are less supportive of vaccination policies. McMahon et al. (2020) conducted a research study on the knowledge of autism in the general population. Participants in the highest quartile underestimated their knowledge and overestimated the knowledge of other participants. Bias was also found in research from Nyhan and Reifler (2015), which examined the extent of misperception of the seasonal flu vaccine. Dunning et al. (2004) wrote that people tend to be overly optimistic about health risks and show pluralistic ignorance in ways that affect their health-related behaviors. They also show confidence in their self-diagnosis as a result of the double curse of incompetence, which we explained earlier in this paper (Dunning et al., 2004).

The Dunning-Kruger effect also has criticism. Krueger and Mueller (2002) state that the Dunning-Kruger effect is due to statistical regression and better-than-average heuristics. Where a better-than-average effect is present, poor performers make larger errors, and any increase in better-than-average effect increases regression and asymmetry of errors. Burson et al. (2006) develop a critique and propose that the Dunning-Kruger effect can be explained by the noise-plus-bias model. It means that people at all levels of performance are equally poor at assessing their relative performance. They overestimate themselves at simple tasks and underestimate themselves at difficult tasks. Plohl and Musil (2018) in their research (Study 1) in the field of grammar of undergraduate students in Slovenia, conclude that, despite the task being difficult, poor performers overestimated their results the most, while top performers were the most accurate in assessing their knowledge. These findings are inconsistent with Burson et al. (2006). Burson et al. (2006) also add that in Dunning-Kruger effect studies, participants are often without feedback when, in a real-world environment, an individual quickly receives feedback, e.g., student’s grade. Kruger and Dunning (2002) state that regression alone cannot explain why the low-skilled are unaware. Ehrlinger et al. (2008) conducted studies in which they corrected measurement errors and sought a link between reality and perception in the case of fully reliable instruments of performance and perception assessment. They find that the changes are minor.

Krajč and Ortmann (2008) suggest that the unqualified have a much more difficult problem of reasoning or a signal extraction problem that is different for the unskilled and capable. Low performers have more difficulty estimating their percentile because there are more of them than there are top performers. Low performers are at the bottom, so their errors on self-assessment move up as they cannot underestimate themselves. Because there are fewer top performers, they do not nullify what’s going on at the bottom, so most of the errors that remain for the observer are the overestimations of the poor performers. Schlösser et al. (2013) reject these criticisms in their research, however, adding that the Krajč-Ortmann model provided a better picture of the underestimation of top performers. Pennycook et al. (2017) find that part of the reason for people’s bias is a lack of awareness of their own bias or indifference to it. Intuitive individuals significantly overestimated their performance on the cognitive reflection test; among more analytical individuals, this phenomenon decreased and eventually reversed. The authors add that the results cannot be attributed to regression towards the mean alone.

## 2 Materials and methods

We conducted two studies to examine the *primus inter pares effect* or *better-than-average effect (illusory superiority)* among high school graduates in Slovenia. In study 1, participants self-assessed 16 traits or abilities on a scale from 1 to 99 in comparison to other peers (questionnaire in Appendix A). The traits and abilities used in our study were derived from the research of Leskovar and Baggia (2021), in which participants (n = 1,750) answered questions about their competencies in the tool that was used to help final year high school students choose future study programs. We chose traits and abilities from five areas: teamwork, interpersonal skills, emotional intelligence, problem solving, and decision making. Traits and abilities from these areas had the highest Cronbach’s Alpha coefficient (from 0.72 to 0.83). In the mentioned research, participants assessed the level of their competencies as mostly positive, with an average between $\bar{x}$ = 0.66 and $\bar{x}$ = 0.88. It should be noted that it is possible that the majority of participants possess a high level of those competencies. But in a certain group, in our case, last year high school students, the majority cannot be above average. In our study, in which 264 high school graduates participated, we used only complete data. We sent an online questionnaire to high schools across Slovenia and asked them to forward it to their students. Participation was voluntary and anonymous.

In study 2, we used a 360-degree method with one class of high school graduates. Each participant assessed three traits and abilities from the area of interpersonal skills in a questionnaire for themselves and for each of their fellow students in the class. A total of 14 high school graduates were present in class at the time of study. To ensure anonymity, we assigned each participant a number, which we wrote on the board in the class along with their name. That way, they knew which classmate they assessed under each number, and we asked them to circle the number under which they assessed their own traits and skills. After the assessment was done, we erased the code list from the board, and the data became anonymous. Some participants did not complete assessments for every other member of the group nevertheless, we used the data from the ones they did assess.

## 3 Results

### 3.1 Study 1

First results show that the mean of self-assessment of every ability and trait is above average, ranging from the lowest $\bar{x}$ = 59.98 (SD = 20.860) for obtaining all possible information before making a decision, to highest $\bar{x}$ = 72.95 (SD = 19.566) for the effort in the conversation to make the interlocutor feel considered. We investigated further with one sample t-test, in which we set the test value first to 55 t(263), and later to 60 t(263). At test value 55 t(263), the statistical significance for all abilities and traits was *p* < 0.001. At test value 60 t(263), there was statistical significance for half of the abilities and traits; the one aforementioned with the highest mean resulted in t(263) = 10.755, *p* < 0.001. In each area, at least one ability or trait had statistical significance. Internal consistency results show the highest Cronbach’s Alpha of 0.78 for problem solving, followed by 0.75 for interpersonal skills, both of which are acceptable. Rest areas have a Cronbach’s Alpha between 0.60 and 0.65 which is considered questionable internal consistency. Percentiles show that the answers were considerably ticked to positive side. At the point of the 25th percentile, the ability or trait with the lowest percentile was recognizing your own emotions, reaching 46th percentile, while highest was the effort in the conversation to make the interlocutor feel considered reaching 61st percentile. The majority of traits and abilities reached the 50th percentile, which we would consider average (although we could argue that average would be 40th-60th percentile). At the point of the 50th percentile, the participants rated themselves from the 60th to the 76th percentile, and at the point of the 75th percentile, the deviation becomes smaller, ranging from the 75th to the 90th percentile. Statistics are presented in Table 1.

**Table 1:** Descriptive statistics and one sample *t*-tests.

	M	SD	Percentile					
			25	50	75	CA	TV 55: t(263)	TV 60: t(263)
Coordination with others	60.81	18.494	50	60	75	0.599	5.102^***^	0.709
Cooperation in the group	60.31	20.192	50	61	75		4.276^***^	0.253
Accepting a different opinion	67.61	21.403	50	70	82.75		9.576^***^	5.780^***^
Sharing ideas with others	60.18	22.283	48.50	60	76.75		3.778^***^	0.133
Promoting a positive atmosphere and good relations	70.03	21.403	55.75	75	85.75	0.750	11.413^***^	7.617^***^
The effort in the conversation to make the interlocutor feel considered	72.95	19.566	61	75	88.75		14.907^***^	10.755^***^
Resolving a dispute if it arises	64.82	20.772	50	65	80		7.680^***^	3.769^***^
Attention to the emotions of others	72.92	20.626	58	76	89.75	0.646	14.117^***^	10.178^***^
Understanding why a person expresses certain emotions	68.16	19.844	53.50	70	81		10.778^***^	6.684^***^
Recognizing your own emotions	60.53	22.914	46	61.50	75.75		3.921^***^	0.376
Recognizing the cause of the problem	61.59	19.064	50	60	75	0.788	5.621^***^	1.359
Recognizing other paths in challenges	61.61	19.341	50	61	75		5.550^***^	1.349
Recognizing better solutions to problems	63.13	17.869	50	61	75.75		7.395^***^	2.848^**^
Consideration of all options before making a decision	62.39	21.622	50	62	80	0.636	5.553^***^	1.796
Obtaining all possible informations before making a decision	59.98	20.860	49	60	75		3.883^***^	−0.012
Persistence in the decision taken	67.01	21.491	50	69.50	82		9.078^***^	5.298^***^

*p < 0.05; **p < 0.01; ***p < 0.001.

**Figure 1:**
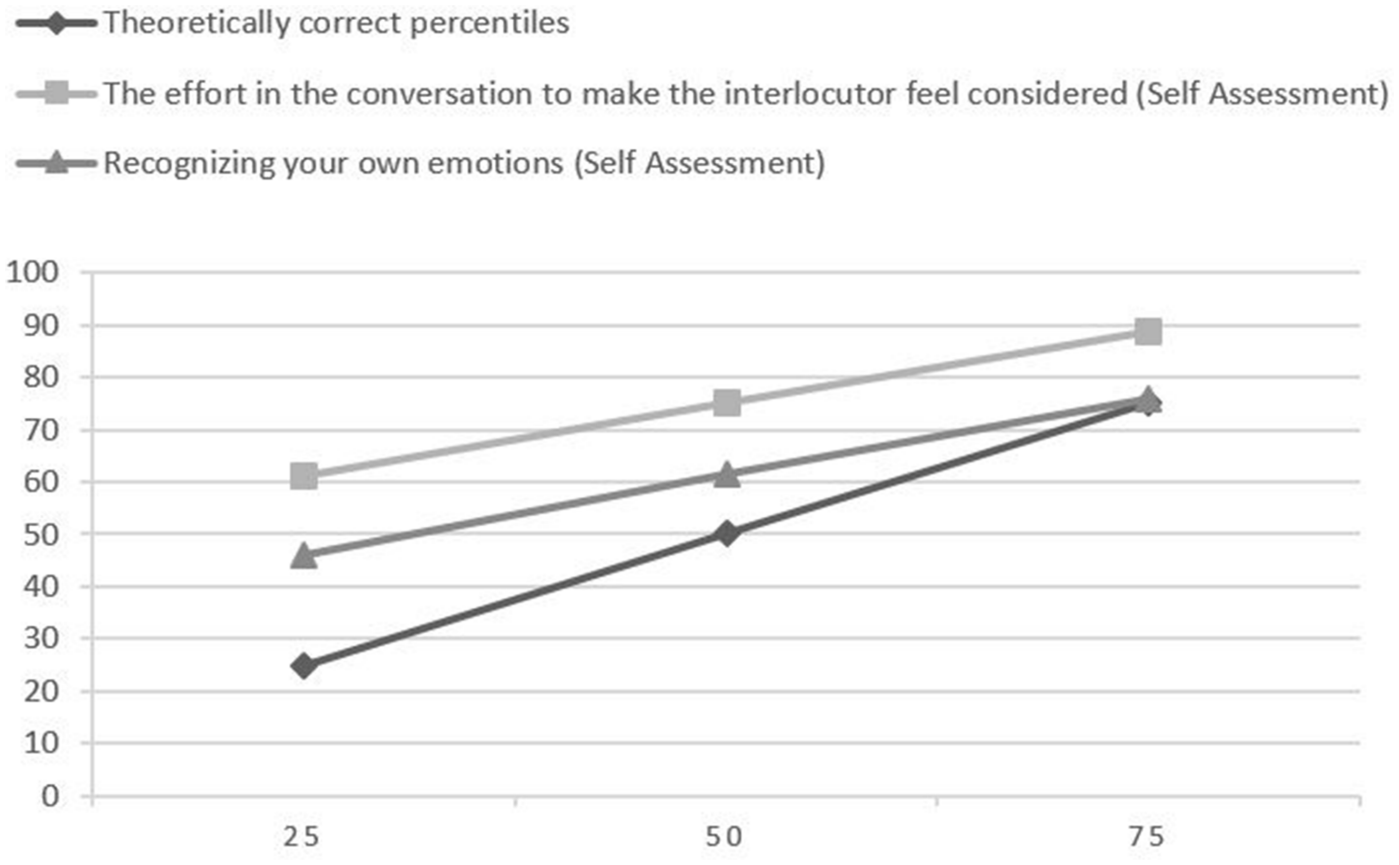
Deviation from theoretically correct percentiles.

In Figure 1, we visualized comparison of the traits or abilities with the lowest and highest percentiles in comparison to the theoretically correct percentiles.

**Figure 2:**
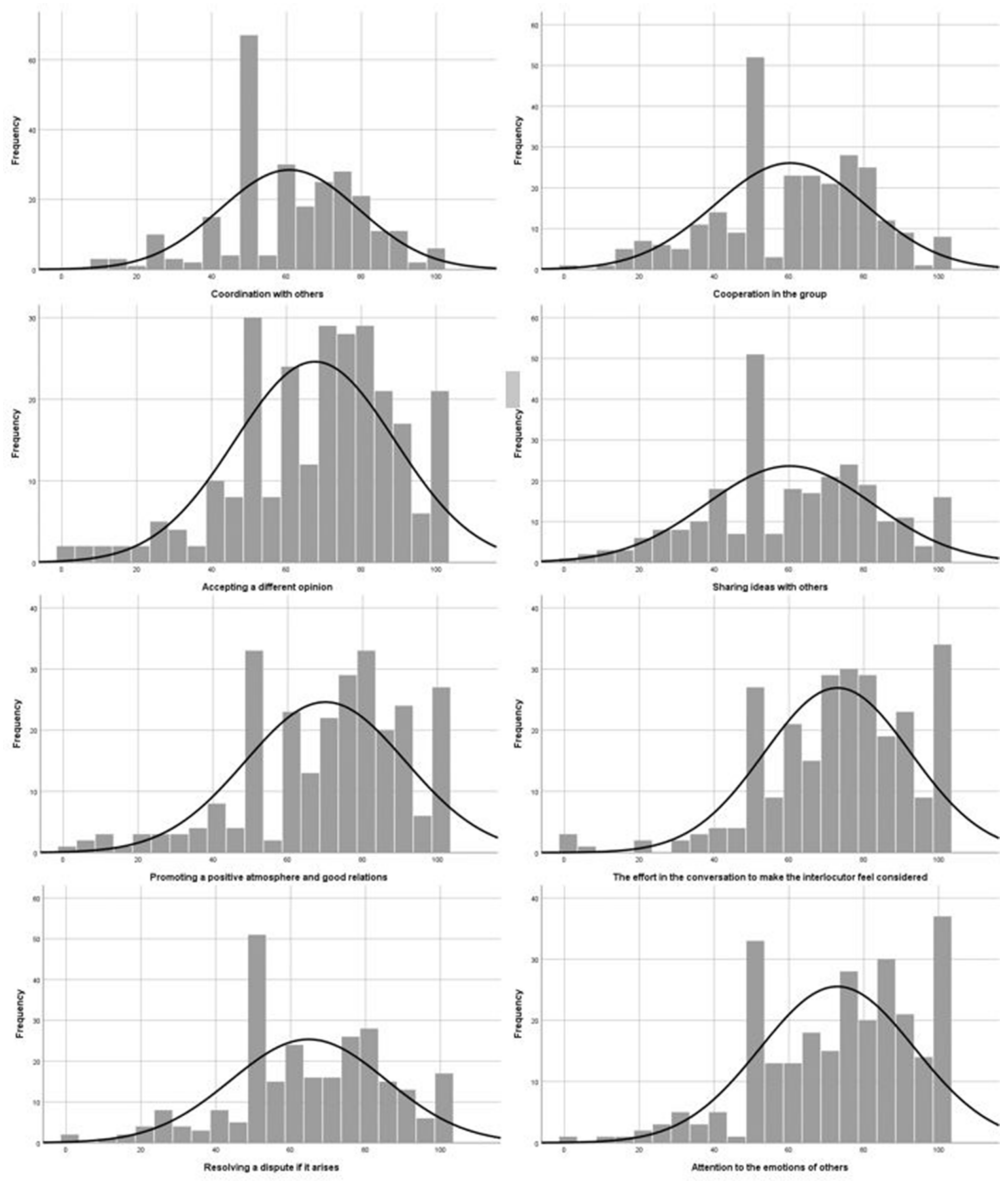
Histograms for self-assessment of abilities and traits 1/2.

**Figure 3:**
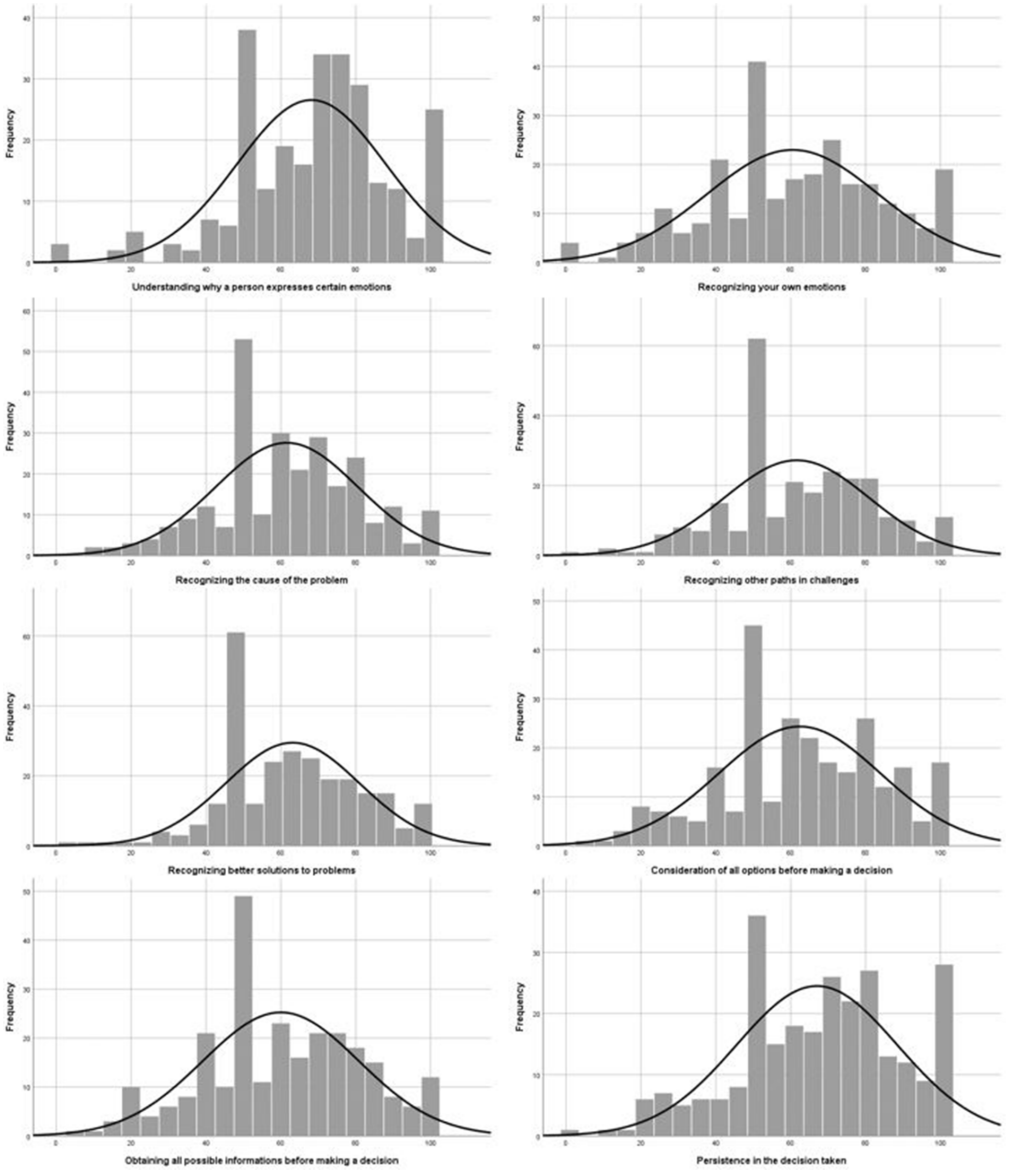
Histograms for self-assessment of abilities and traits 2/2.

We investigated distribution of data for each of the 16 abilities and traits with histograms. As Figures 2, 3 show, the most common answer was 50, which means average, which can be attributed to indecisiveness (for more about indecisiveness, look at Frost and Shows, 1993). Still, we can see that self-assessment for all traits and abilities was considerably ticked to positive side.

**Figure 4:**
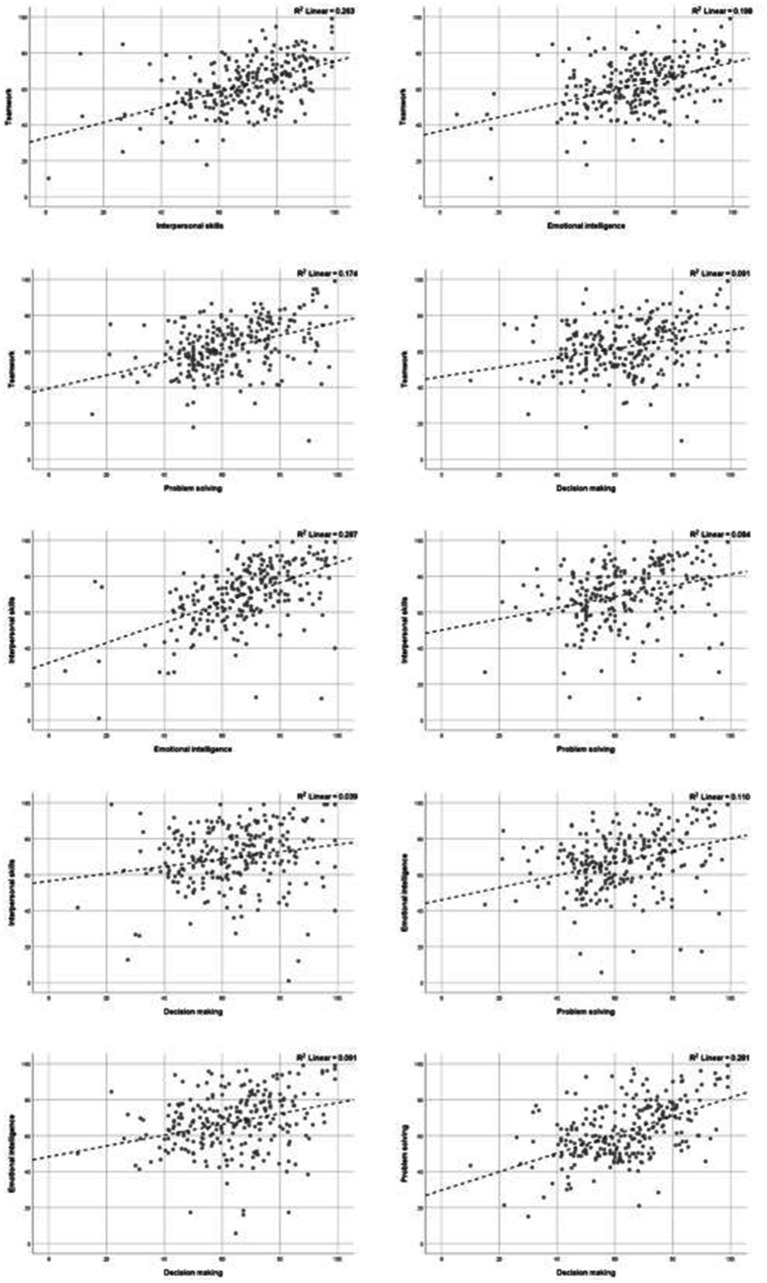
Correlation scatterplots of ability and trait areas.

We further investigated if self-overestimation in one area of abilities and traits correlates with self-overestimation in another area. Results show a weak to moderate positive correlation (Table 2). The highest correlation is between *teamwork* and *interpersonal skills*, r = 0.513, *p* < 0.001; while the lowest is between *interpersonal skills* and *decision making* r = 0.196, *p* < 0.01. Figure 4 presents correlation scatterplots for each pair of areas of abilities or traits.

**Table 2:** Pearson correlation.

	Teamwork	Interpersonal skills	Emotional intelligence	Problem solving	Decision making
Teamwork	1	0.513^***^	0.445^***^	0.417^***^	0.301^***^
Interpersonal skills	0.513^***^	1	0.536^***^	0.290^***^	0.196^**^
Emotional intelligence	0.445^***^	0.536^***^	1	0.331^***^	0.301^***^
Problem solving	0.417^***^	0.290^***^	0.331^***^	1	0.531^***^
Decision making	0.301^***^	0.196^**^	0.301^***^	0.531^***^	1

*p < 0.05; **p < 0.01; ***p < 0.001.

### 3.2 Study 2

**Figure 5:**
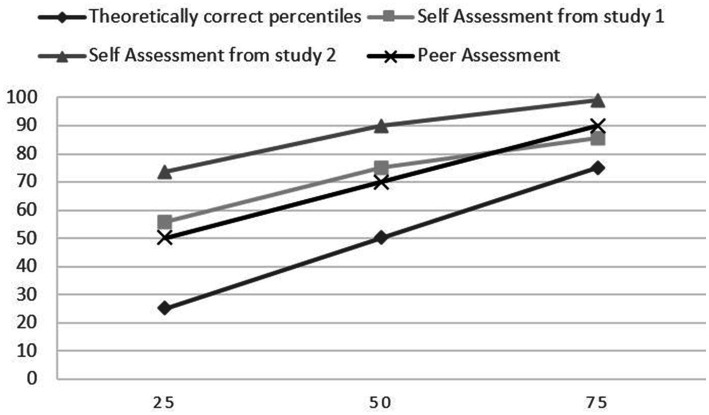
Comparison of percentiles of promoting a positive atmosphere and good relations.

**Figure 6:**
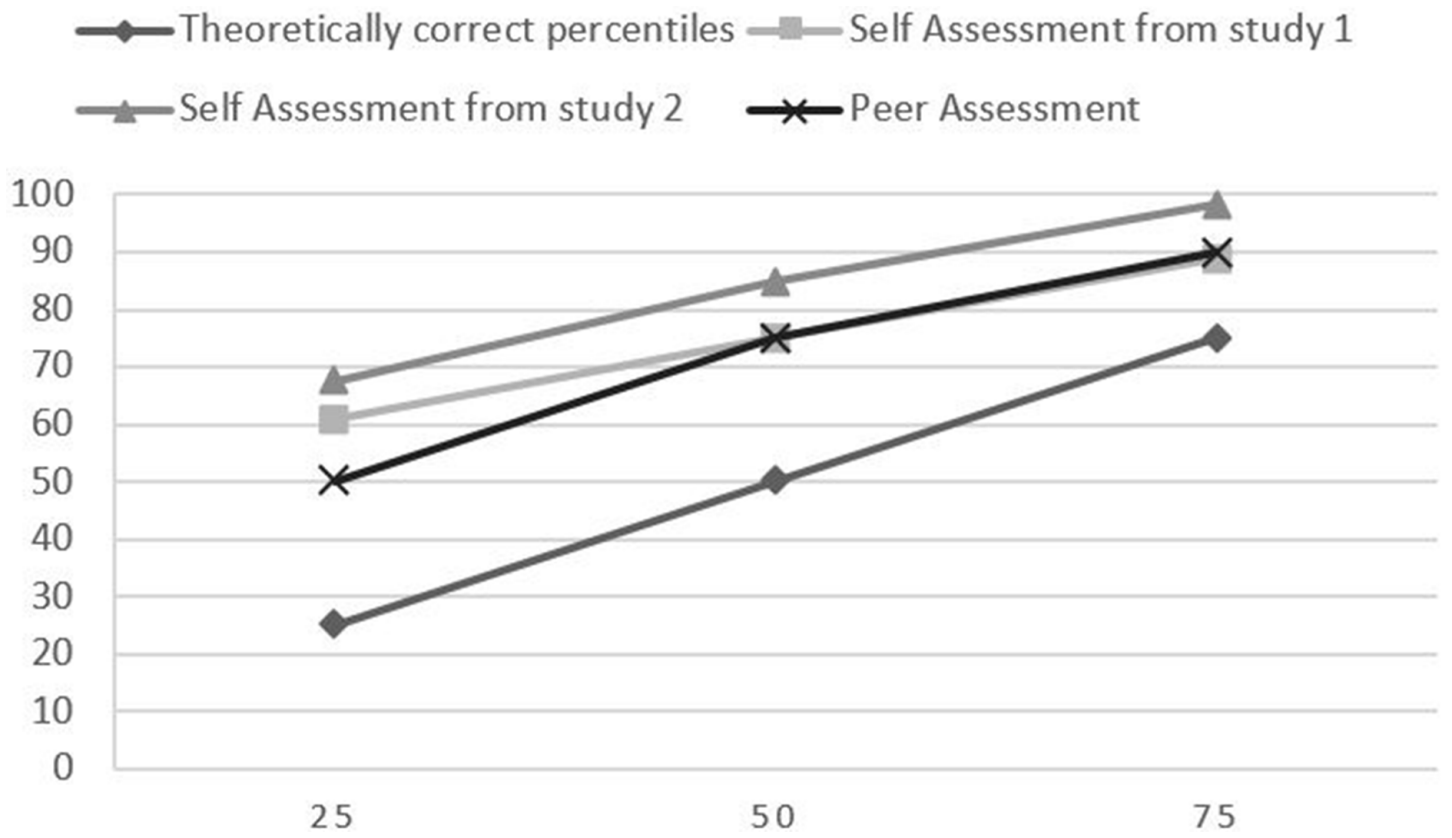
Comparison of percentiles of the effort in the conversation to make the interlocutor feel considered.

**Figure 7:**
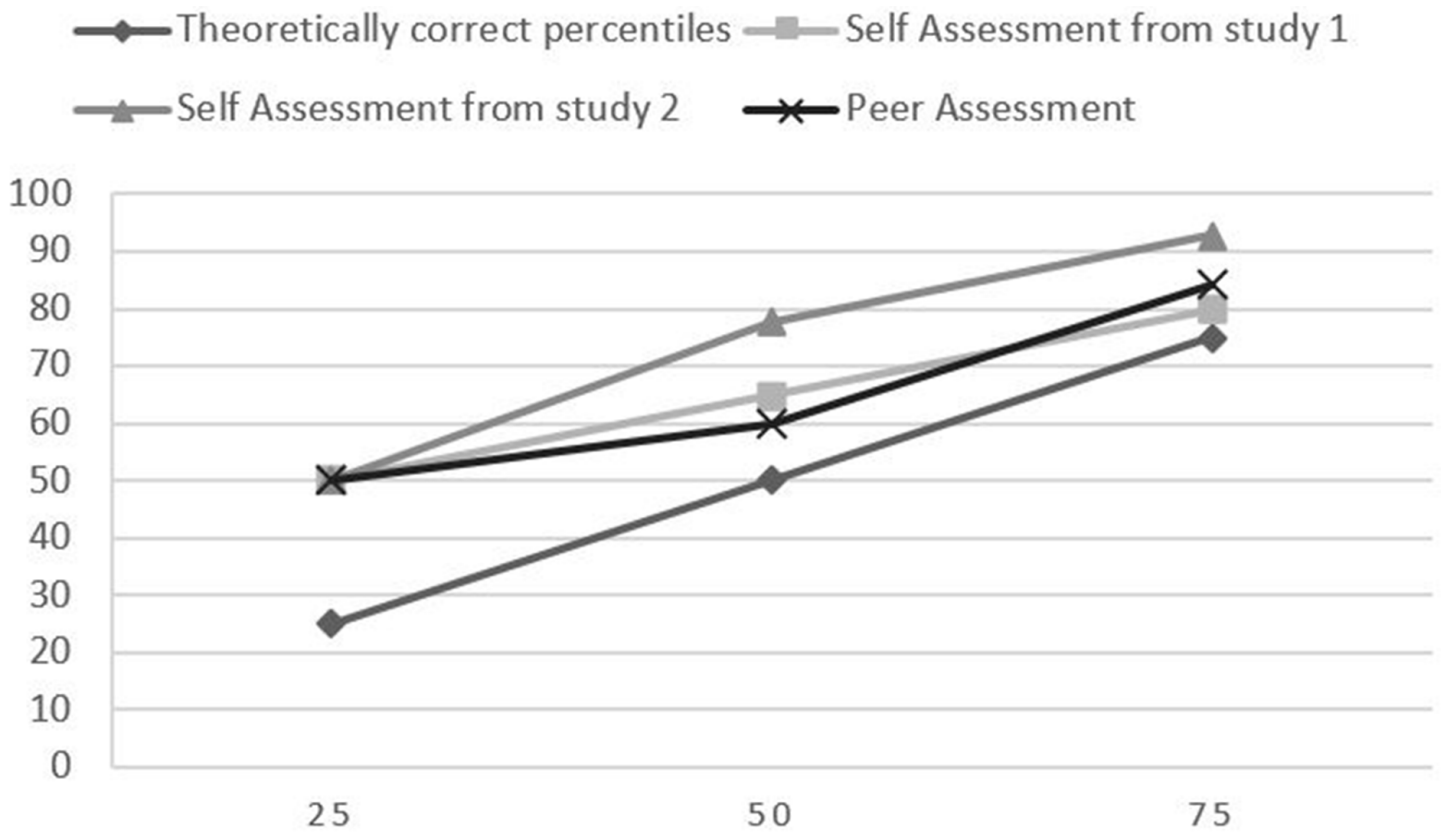
Comparison of percentiles of resolving a dispute if it arises.

We used traits and abilities from the area of interpersonal skills because they had the highest mean and percentiles (look at Table 1), and they also had the second highest Cronbach’s Alpha = 0.75. 360-degrees method was used in Study 2. Students completed a self-assessment for three abilities or traits in comparison to their classmates, and they assessed each of their classmates for the same traits or abilities. The results in Table 4 show that self-assessment was higher than peer assessment on average in each of the three traits. 14 participants and three traits amounted to a total of 42 self-assessments, and only 4 rated themselves below average. While peer-assessment was lower, still only seven means were below average from a total of 42 means (Table 3). The main difference observed between self-assessment and peer assessment is that self-assessment was on average way higher than peer assessment. We compared the data from both studies for the three traits or abilities that were used in studies 1 and 2. For all three traits or abilities, the means were lower from peer assessment than from self-assessments in both studies 1 and 2. Figures 5–7 present the difference between percentiles of self-assessments from both studies, peer assessment and theoretically correct percentiles.

**Table 3:** Descriptive statistics for self-assessment and peer assessment.

	Promoting a positive atmosphere and good relations				The effort in the conversation to make the interlocutor feel considered				Resolving a dispute if it arises			
ID	SA	Peer assessment			SA	Peer Assessment			SA	Peer assessment		
		N	M	SD		N	M	SD		N	M	SD
1	99	13	63.85	27.248	99	13	70.69	30.396	70	13	65.62	31.170
2	90	13	75.92	21.635	90	13	66.77	24.403	50	13	60.23	29.814
3	50	13	58.46	28.462	60	13	63.15	24.300	30	13	60.62	27.657
4	99	12	49.17	34.766	99	12	50.83	36.799	99	12	52.00	35.396
5	90	13	55.00	27.842	98	13	68.54	28.884	91	13	61.77	28.688
6	99	13	76.00	17.753	70	13	72.23	22.953	75	13	72.15	19.222
7	99	13	83.23	15.605	99	13	80.77	18.404	99	13	73.38	17.600
8	80	13	83.31	16.347	40	13	82.54	19.125	50	13	77.38	25.155
9	40	13	55.77	34.150	60	13	59.08	33.428	30	13	55.92	29.432
10	90	14	72.71	26.357	80	14	72.21	27.344	90	14	69.36	27.433
11	80	14	64.21	33.368	80	14	66.71	30.560	80	14	64.21	28.767
12	75	13	39.31	28.889	80	13	44.23	37.745	60	13	41.85	34.019
13	70	13	62.31	29.767	90	13	64.92	29.127	99	13	61.38	22.556
14	95	13	36.23	27.365	90	13	48.85	37.318	80	13	37.77	29.942

SA, Self-Assessment; N, Sample size; M, Mean; SD, Standard Deviation.

**Table 4:** Means and percentiles from study 1 and study 2.

	SA1 (N = 264)					SA2 (N = 14)					PA (N = 196)				
	M	SD	Percentile			M	SD	Percentile			M	SD	Percentile		
			25	50	75			25	50	75			25	50	75
Promoting a positive atmosphere and good relations	70.03	21.403	55.75	75.00	85.75	82.57	18.608	73.75	90.00	99.00	63.09	29.172	50.00	70.00	90.00
The effort in the conversation to make the interlocutor feel considered	72.95	19.566	61.00	75.00	88.75	81.07	17.955	67.50	85.00	98.25	65.67	29.648	50.00	75.00	90.00
Resolving a dispute if it arises	64.82	20.772	50.00	65.00	80.00	71.64	24.260	50.00	77.50	93.00	61.39	28.702	50.00	60.00	84.25

SA1, Self-Assessment from study 1; SA2, Self-Assessment from study 2; PA, Peer Assessment from study 2; M, Mean; SD, Standard Deviation.

## 4 Discussion

Results of our research indicate a *primus inter pares* effect or better-than-average effect (or illusory superiority) among the high school graduates in Slovenia. Results for traits from the area of interpersonal skills show that even peer assessment was relatively high, though it was in the majority lower than self-assessment, which confirms the claims of Codol (1975); Alicke et al. (1995); and Pedregon et al. (2012) that people usually assess more generously individuals they know or have contact with. All traits have a positive connotation, so it falls in line with Brown (2012) who states the bias is greater for traits that participants deem more important. Brown (2012) suggests in his study that motivation influences bias; similarly, Alicke et al. (1995) state that individuals judge themselves more favorably than others because of heuristic and functions as a means of maintaining self-confidence. Study 1 also suggests indecisiveness of individuals; it could be debated that this is not due to indecisiveness but rather to heuristics, but the latter would probably yield more positive assessments. As Burson et al. (2006) noted for Dunning-Kruger effect studies, participants are in a controlled environment and often without feedback, while in a real-world environment they quickly receive feedback. We argue that this is true to a certain extent. For example, if we asked a high school graduate’s class about math knowledge, we believe that overestimation would be lower due to the fact that they have attended the class for a few years, know each other, and most importantly, have been receiving feedback all this time. If an individual has been constantly receiving a bad math grade, while his classmates have been receiving good grades, then he would probably assess his math skill as aligned with or more aligned with that feedback. Now the question is, what if an individual receives feedback but does not see the feedback for other classmates? He knows he is bad at math but does not know how good his classmates are, so his self-overestimation would most likely be higher. Like Kruger and Dunning (1999); Dunning et al. (2003); and Dunning (2011) explained with double curse of incompetence. We present a question for further investigation: who has a bigger bias? For example, for the ability of resolving a dispute if it arises, does the individual who rarely or never gets into conflicts have a bigger bias than the individual who is often in conflicts? The person who is never or rarely in conflict may think that he is good at resolving conflicts because he rarely meets with such situations, which as Weinstein (1987) stated, provokes unrealistic optimism. On the other hand, the person who is often in conflict may believe that he is good at solving them because he frequently finds himself in such situations and has experience with them. Of course, the latter also depends on the feedback the individual receives. *Primus inter pares* effect or better-than-average effect cannot be attributed to only one factor but several, such as motivation, heuristics, double curse of incompetence mostly associated with the Dunning-Kruger effect, feedback, not understanding the meaning of certain competencies, information deficit, and neglecting information; similarly, as Dunning et al. (2004); Brown (2012) and Alicke et al. (1995) stated. We should also ask ourselves what do participants in studies consider as average, perhaps average has a bit of a negative connotation and is actually considered below average in a sense. Or an individual who does not possess certain abilities or traits would believe that neither do the majority of people, so he would place his level of ability as average. We also must take into account that the bias is not static. The individual who is biased in one area is not necessarily biased in other areas as well. Some people noted that the researchers are biased and that the results are greatly dependent on the methods they use. Of course, this is true to some extent. For example, in Dunning-Kruger effect studies, if we ask a participant about his knowledge of geography, and let us say that the individual is actually good at geography, but we use five questions from the area of geography, those five questions are his weak spots. If we use different five questions, he might be much better or much worse. So to some extent, researchers do influence the outcome with their methods. Nevertheless, along with criticism of statistical methods used in Dunning-Kruger effect studies and *primus inter pares* effect (better-than-average effect) studies, the question still remains: why do results, when put in context, still show such bias? Let us assume for a moment that it is all due to wrong statistical methods, etc., then why do we still get results that people with the least knowledge about autism connect autism with vaccines the most (look at Motta et al., 2018; McMahon et al., 2020); why did the candidates who failed the test largely overestimate their driving skills (look at Mynttinen et al., 2009); and so on, for various competencies and traits across various domains that we presented in the literature review in this paper. Also, authors do not always use the same measurements and statistical methods, and yet the bias persists. Krajč and Ortmann (2008) stated that low performers cannot underestimate themselves (mostly) because they are at the bottom and so their errors move only up; the opposite holds for top performers (this mathematical verity was also stated by Kruger and Dunning, 1999). Krajč and Ortmann (2008) add that because there are fewer top performers, they do not nullify the errors of low performers for the observer. But this is the crux of the problem: There are more low performers who overestimate their abilities. Even if we ignore the immediate dangers that can occur due to biases, the consequences can be lasting and widespread across entire societies. For example, Roediger et al. (2019) assessed the knowledge of people from 11 countries (n = 1,338) about which country contributed the most to victory in World War II. Participants from several countries believe that it was their country. While we can agree that all countries contributed, it would be wrong not to acknowledge that the Soviet Union battled the vast majority of the Axis’s army. Misinformation can be very dangerous as it can shape certain knowledge, although false, to be desirable. One example of wider consequences due to overestimation, presented by Hall (1982), is the building of Sydney’s Opera House, which was initially estimated in 1957 to cost 7 million Australian dollars and to be completed by 1963 but was actually finished 10 years later at the cost of 102 million Australian dollars. Major problems were engineering design, inadequate cost estimates, and technical control. Of course, it would be almost impossible to entirely eliminate “negative bias” because, as mentioned before, it is not static and is subject to deterministic nonperiodic flow (look at Lorenz, 1963; and term Chaos Theory), but we should still strive to minimize it. To put our results in the context of this discussion, we can see that most overestimation is in the area of interpersonal skills, which in comparison to other areas is more of a social skill area and harder to measure than, for example, decision making or problem solving. Also, one of the reasons could be the lower or inadequate feedback that we mentioned earlier. For example, one probably receives more feedback over the course of high school in decision making, problem solving, and teamwork than in interpersonal skills, as those can directly impact grades while interpersonal skills less so. Of course, that would leave the question of emotional intelligence, which is also more of a social skill area, but even in that area, two abilities or traits have higher overestimations on par with the ones in interpersonal skills. People generally socialize with each other in high school, either with friends or classmates, so as these social traits and abilities are desirable, people tend to overestimate their level of them. As seen, for example, in the study of moral superiority by Tappin and McKay (2016), although their study shows that in the case of moral superiority it is not related to self-esteem, while elsewhere it typically is. We should strive to reduce bias as much as possible and find ways, techniques, and methods to do so.

## 5 Conclusion and limitations

Across various research studies, results show that people are affected by cognitive biases. To some extent, it is positive and serves as a survival mechanism, like motivation to carry out certain actions and achieve goals, or to satisfy curiosity and create innovations. To another extent, it is negative as it poses danger to oneself, an organization, or even society. While one might not think that bias can be so dangerous, it can. For example, on a societal level, as Machiavelli (2014) wrote, people often wish to change the government at all costs, thinking that change will be for the better, but in the majority, it worsens the situation. Our paper contributes a small insight into illusory superiority among high school graduates in Slovenia, which confirms that individuals overestimate their positive traits and abilities in comparison to others and assess the traits and abilities of people they know or have contact with more generously. We found out that self-assessment was way higher than peer assessment, both however point out overestimation. Suggested reasons for overestimation of those abilities and traits are the desirability of these social skills and traits and lower, inadequate feedback and better-than-average heuristic. Studies also suggest indecisiveness of high school graduates. Overestimation can occur due to various reasons, such as motivation, lack of feedback, neglect of feedback, neglect and deficit of information, misinformation, heuristics, and the double curse of incompetence as defined by Kruger and Dunning (1999). We should all strive to minimize biases, so it is of paramount importance to derive methods for doing so. The limitations of our studies were the small sample in the second study and measurements of other factors like self-esteem, intuition, or motivation to investigate sources or links for overestimation. For further research, we suggest adding techniques that would try to guide participants to more accurate self-assessments and measure the success of those techniques. More longitudinal studies should be conducted for tracking biases, its causes and consequences. Schools should introduce critical thinking skills in the curriculum to help recognizing potential biases.

## Data Availability

The raw data supporting the conclusions of this article will be made available by the authors, without undue reservation.

## References

[ref1] Akhmetshin E. M. Mueller J. E. Yumashev A. V. Kozachek A. V. Prikhodko A. N. Safonova E. E. (2019). Acquisition of entrepreneurial skills and competences: curriculum development and evaluation for higher education. J. Entrep. Educ. 22, 1–12.

[ref2] Alicke M. D. Klotz M. L. Breitenbecher D. L. Yurak T. J. Vredenburg D. S. (1995). Personal contact, individuation, and the better-than-average effect. J. Pers. Soc. Psychol. 68, 804–825. doi: 10.1037/0022-3514.68.5.804

[ref3] Austin Z. Gregory P. A. M. Galli M. (2008). “I just don’t know what I’m supposed to know”: evaluating self-assessment skills of international pharmacy graduates in Canada. Res. Soc. Adm. Pharm. 4, 115–124. doi: 10.1016/j.sapharm.2007.03.002 18555965

[ref4] Bell P. Volckmann D. (2011). Knowledge surveys in general chemistry: confidence, overconfidence, and performance. J. Chem. Educ. 88, 1469–1476. doi: 10.1021/ed100328c

[ref5] Bergquist M. (2020). Most people think they are more pro-environmental than others: a demonstration of the better-than-average effect in perceived pro-environmental behavioral engagement. Basic Appl. Soc. Psychol. 42, 50–61. doi: 10.1080/01973533.2019.1689364

[ref6] Brown J. D. (2012). Understanding the better than average effect: motives (still) matter. Personal. Soc. Psychol. Bull. 38, 209–219. doi: 10.1177/0146167211432763 22205623

[ref7] Bunay R. Siguenza W. Flores K. Serpa-Andrade L. (2017). “Impact of the Dunning Kruger effect on psychology students at the University of Cuenca” in Advances in human factors in training, education, and learning sciences. Advances in Intelligent Systems and Computing, 596. ed. Andre T. (Cham: Springer), 383–391.

[ref8] Burson K. A. Larrick R. P. Klayman J. (2006). Skilled or unskilled, but still unaware of it: how perceptions of difficulty drive Miscalibration in relative comparisons. J. Pers. Soc. Psychol. 90, 60–77. doi: 10.1037/0022-3514.90.1.60 16448310

[ref9] Codol J.-P. (1975). On the so-called “superior conformity of the self” behavior: twenty experimental investigations. Eur. J. Soc. Psychol. 5, 457–501. doi: 10.1002/ejsp.2420050404

[ref10] Coutinho M. V. C. Thomas J. Alsuwaidi A. S. M. Couchman J. J. (2021). Dunning-Kruger effect: intuitive errors predict overconfidence on the cognitive reflection test. Front. Psychol. 12, 1–10. doi: 10.3389/fpsyg.2021.603225, PMID: 33897524 PMC8060648

[ref11] Darwin C. (1871). The descent of man, and selection in relation to sex. London: John Murray.

[ref12] Dunning D. (2011). The Dunning-Kruger Effect. Adv. Exp. Soc. Psychol. 44, 247–296. doi: 10.1016/b978-0-12-385522-0.00005-6

[ref13] Dunning D. (2015). On identifying human capital: flawed knowledge leads to faulty judgments of expertise by individuals and groups. Adv. Group Process. 32, 149–176. doi: 10.1108/S0882-614520150000032006

[ref14] Dunning D. Heath C. Suls J. M. (2004). Flawed self-assessment. Psychol. Sci. Public Interest 5, 69–106. doi: 10.1111/j.1529-1006.2004.00018.x 26158995

[ref15] Dunning D. Johnson K. Ehrlinger J. Kruger J. (2003). Why people fail to recognize their own incompetence. Curr. Dir. Psychol. Sci. 12, 83–87. doi: 10.1111/1467-8721.01235

[ref16] Ehrlinger J. Johnson K. Banner M. Dunning D. Kruger J. (2008). Why the unskilled are unaware: further explorations of (absent) self-insight among the incompetent. Organ. Behav. Hum. Decis. Process. 105, 98–121. doi: 10.1016/j.obhdp.2007.05.002 19568317 PMC2702783

[ref17] Feld J. Sauermann J. de Grip A. (2017). Estimating the relationship between skill and overconfidence. J. Behav. Exp. Econ. 68, 18–24. doi: 10.1016/j.socec.2017.03.002

[ref18] Frost R. O. Shows D. L. (1993). The nature and measurement of compulsive indecisiveness. Behav. Res. Ther. 31, 683–IN2. doi: 10.1016/0005-7967(93)90121-a 8216169

[ref19] Hall P. (1982). Great planning disasters. Berkeley, CA: University of California Press.

[ref20] Horswill M. S. Waylen A. E. Tofield M. I. (2004). Drivers’ ratings of different components of their own driving skill: a greater illusion of superiority for skills that relate to accident involvement. J. Appl. Soc. Psychol. 34, 177–195. doi: 10.1111/j.1559-1816.2004.tb02543.x

[ref21] Krajč M. Ortmann A. (2008). Are the unskilled really that unaware? An alternative explanation. J. Econ. Psychol. 29, 724–738. doi: 10.1016/j.joep.2007.12.006

[ref22] Krueger J. Mueller R. A. (2002). Unskilled, unaware, or both? The better-than-average heuristic and statistical regression predict errors in estimates of own performance. J. Pers. Soc. Psychol. 82, 180–188. doi: 10.1037/0022-3514.82.2.180 11831408

[ref23] Kruger J. (1999). Lake Wobegon be gone! The “below-average effect” and the egocentric nature of comparative ability judgments. J. Pers. Soc. Psychol. 77, 221–232. doi: 10.1037/0022-3514.77.2.221, PMID: 10474208

[ref24] Kruger J. Dunning D. (1999). Unskilled and unaware of it: how difficulties in recognizing one’s own incompetence lead to inflated self-assessments. J. Pers. Soc. Psychol. 77, 1121–1134. doi: 10.1037/0022-3514.77.6.1121, PMID: 10626367

[ref25] Kruger J. Dunning D. (2002). Unskilled and unaware--but why? A reply to Krueger and Mueller (2002). J. Pers. Soc. Psychol. 82, 189–192. doi: 10.1037//0022-3514.82.2.189 11831409

[ref26] Leskovar R. Baggia A. (2021). *Data analysis of support tool to choose a profession “Kambi”*. Information Society 2021, Ljubljana, Slovenia.

[ref27] Lorenz E. N. (1963). Deterministic nonperiodic flow. J. Atmos. Sci. 20, 130–141. doi: 10.1175/1520-0469(1963)020<0130:DNF>2.0.CO;2

[ref28] Machiavelli N. (2014). The prince. London: Penguin Classics, Penguin Books.

[ref29] Martinussen L. M. Møller M. Prato C. G. (2017). Accuracy of young male drivers’ self-assessments of driving skill. Transport. Res. F: Traffic Psychol. Behav. 46, 228–235. doi: 10.1016/j.trf.2017.03.001

[ref30] McMahon C. M. Stoll B. Linthicum M. (2020). Perceived versus actual autism knowledge in the general population. Res. Autism Spectr. Disord. 71, 101499–101497. doi: 10.1016/j.rasd.2019.101499

[ref31] Mojzisch A. Elster C. Germar M. (2021). People perceive themselves to adhere more strictly to COVID-19 guidelines than others. Psychol. Health Med. 27, 325–332. doi: 10.1080/13548506.2021.1906435 33779442

[ref32] Motta M. Callaghan T. Sylvester S. (2018). Knowing less but presuming more: Dunning-Kruger effects and the endorsement of anti-vaccine policy attitudes. Soc. Sci. Med. 211, 274–281. doi: 10.1016/j.socscimed.2018.06.032 29966822

[ref33] Mynttinen S. Sundström A. Vissers J. Koivukoski M. Hakuli K. Keskinen E. (2009). Self-assessed driver competence among novice drivers – a comparison of driving test candidate assessments and examiner assessments in a Dutch and Finnish sample. J. Saf. Res. 40, 301–309. doi: 10.1016/j.jsr.2009.04.006, PMID: 19778654

[ref34] Nietzsche F. (1910). Human, all too human: A book for free spirits (Zimmern H., Trans.). Edinburgh: T. N. Foulis.

[ref35] Nowell C. Alston R. M. (2007). I thought I got an a! Overconfidence across the economics curriculum. J. Econ. Educ. 38, 131–142. doi: 10.3200/jece.38.2.131-142

[ref36] Nyhan B. Reifler J. (2015). Does correcting myths about the flu vaccine work? An experimental evaluation of the effects of corrective information. Vaccine 33, 459–464. doi: 10.1016/j.vaccine.2014.11.017, PMID: 25499651

[ref37] Pavel S. R. Robertson M. F. Harrison B. T. (2012). The Dunning-Kruger effect and SIUC University’s aviation students. J. Aviat. Technol. Eng. 2, 125–129. doi: 10.5703/1288284314864

[ref38] Pazicni S. Bauer C. F. (2014). Characterizing illusions of competence in introductory chemistry students. Chem. Educ. Res. Pract. 15, 24–34. doi: 10.1039/c3rp00106g

[ref39] Pedregon C. A. Farley R. L. Davis A. Wood J. M. Clark R. D. (2012). Social desirability, personality questionnaires, and the “better than average” effect. Personal. Individ. Differ. 52, 213–217. doi: 10.1016/j.paid.2011.10.022

[ref40] Pennycook G. Ross R. M. Koehler D. J. Fugelsang J. A. (2017). Dunning-Kruger effects in reasoning: theoretical implications of the failure to recognize incompetence. Psychon. Bull. Rev. 24, 1774–1784. doi: 10.3758/s13423-017-1242-7, PMID: 28224482

[ref41] Plohl N. Musil B. (2018). Do I know as much as I think I do? The Dunning-Kruger effect, overclaiming, and the illusion of knowledge. Horiz. Psychol. 27, 20–30. doi: 10.20419/2018.27.481

[ref42] Roediger H. L. Abel M. Umanath S. Shaffer R. A. Fairfield B. Takahashi M.. (2019). Competing national memories of World War II. Proc. Natl. Acad. Sci. 116, 16678–16686. doi: 10.1073/pnas.1907992116, PMID: 31405968 PMC6708356

[ref43] Saito K. Trofimovich P. Mariko A. In'nami Y. (2020). Dunning-Kruger effect in second language speech learning: how does self perception align with other perception over time? Learn. Individ. Differ. 79:101849. doi: 10.1016/j.lindif.2020.101849

[ref44] Schlösser T. Dunning D. Johnson K. L. Kruger J. (2013). How unaware are the unskilled? Empirical tests of the “signal extraction” Counterexplanation for the Dunning-Kruger effect in self-evaluation of performance. J. Econ. Psychol. 39, 85–100. doi: 10.1016/j.joep.2013.07.004

[ref45] Sullivan P. J. Ragogna M. Dithurbide L. (2018). An investigation into the Dunning–Kruger effect in sport coaching. Int. J. Sport Exerc. Psychol. 17, 591–599. doi: 10.1080/1612197x.2018.1444079

[ref46] Svenson O. (1981). Are we all less risky and more skillful than our fellow drivers? Acta Psychol. 47, 143–148. doi: 10.1016/0001-6918(81)90005-6

[ref47] Tappin B. M. McKay R. T. (2016). The illusion of moral superiority. Soc. Psychol. Personal. Sci. 8, 623–631. doi: 10.1177/1948550616673878, PMID: 29081899 PMC5641986

[ref48] Tremayne K. S. Newbery G. Tremayne P. Nolan K. A. (2021). Can the Dunning-Kruger effect occur in the motor performance domain? Int. J. Sport Exerc. Psychol. 20, 715–728. doi: 10.1080/1612197X.2021.1929396

[ref49] Ward M. A. Heichelheim F. M. Yeo C. A. (2016). A history of the Roman people. 6th Edn. New York: Routledge.

[ref50] Webb J. A. Karatjas A. G. (2018). Grade perceptions of students in chemistry coursework at all levels. Chem. Educ. Res. Pract. 19, 491–499. doi: 10.1039/c7rp00168a

[ref51] Weinstein N. D. (1987). Unrealistic optimism about susceptibility to health problems: conclusions from a community-wide sample. J. Behav. Med. 10, 481–500. doi: 10.1007/bf00846146, PMID: 3430590

[ref52] Ziegler B. Montplaisir L. (2014). Student perceived and determined knowledge of biology concepts in an upper-level biology course. CBE-Life Sci. Educ. 13, 322–330. doi: 10.1187/cbe.13-09-0175, PMID: 26086662 PMC4041508

