# Supplementary material for: Primus inter pares effect in high schools
Source: Front Psychol. 2024 Oct 11;15:1382062. doi: 10.3389/fpsyg.2024.1382062 (PMC11502392; doi:10.3389/fpsyg.2024.1382062)
Supplement: Supplementary file 1 [file Table_1.docx]

Supplementary Material

# Appendix A

**Self-assessment of traits and abilities in comparison with other 4th year high school students**

The number expresses how much of the peer population you think you are better than. e.g., 50: you rank among the average, you are better than 50% of the peer population, e.g., 75: you rank above average, you are better than 75% of the peer population, e.g., 25: you rank below average, you are better than 25% of the peer population.

***Note: A slider on the interval 1-99 was placed next to statements.**

1. Rate how good you are at: *coordination with others*.
2. Rate how good you are at: *cooperation in the group*.
3. Rate how good you are at: *accepting a different opinion*.
4. Rate how good you are at: *sharing ideas with others*.
5. Rate how good you are at: *promoting a positive atmosphere and good relations.*
6. Rate how good you are at: *the effort in the conversation to make the interlocutor feel considered.*
7. Rate how good you are at: *resolving a dispute if it arises*.
8. Rate how good you are at: *attention to the emotions of others*.
9. Rate how good you are at: *understanding why a person expresses certain emotions*.
10. Rate how good you are at: *recognizing your own emotions*.
11. Rate how good you are at: *recognizing the cause of the problem*.
12. Rate how good you are at: *recognizing other paths in challenges*.
13. Rate how good you are at: *recognizing better solutions to problems*.
14. Rate how good you are at: *consideration of all options before making a decision*.
15. Rate how good you are at: *obtaining all possible information before making a decision*.
16. Rate how good you are at: *persistence in the decision taken*.
